# Non-speculum clinician-taken samples for human papillomavirus testing: a cross-sectional study in older women

**DOI:** 10.3399/BJGP.2021.0708

**Published:** 2022-06-07

**Authors:** Rebecca Landy, Tony Hollingworth, Jo Waller, Laura AV Marlow, Jane Rigney, Thomas Round, Peter D Sasieni, Anita WW Lim

**Affiliations:** Division of Cancer Epidemiology and Genetics, National Cancer Institute, Bethesda, MD, US.; Whipps Cross University Hospital, London, UK.; School of Cancer and Pharmaceutical Sciences, King’s College London, London, UK.; School of Cancer and Pharmaceutical Sciences, King’s College London, London, UK.; School of Cancer and Pharmaceutical Sciences, King’s College London, London, UK.; School of Population Health and Environmental Sciences, King’s College London, London, UK.; School of Cancer and Pharmaceutical Sciences, King’s College London, London, UK.; School of Cancer and Pharmaceutical Sciences, King’s College London, London, UK.

**Keywords:** alphapapillomavirus, cervical cancer screening, cervical intraepithelial neoplasia, diagnostic accuracy, general practice, self-sampling

## Abstract

**Background:**

Cervical cancer incidence and mortality are high in women aged ≥65 years, despite the disease being preventable by screening. Speculum-based screening can become more uncomfortable after the menopause.

**Aim:**

To examine test performance and acceptability of human papillomavirus (HPV) testing on clinician-collected vaginal samples without a speculum (non-speculum).

**Design and setting:**

Cross-sectional study in 11 GP practices and four colposcopy clinics in London, UK, between August 2017 and January 2019.

**Method:**

Non-speculum and conventional (speculum) samples were collected from women aged ≥50 years attending for a colposcopy (following a speculum HPV-positive screening result) or women aged ≥35 years (with confirmed cervical intraepithelial neoplasia (CIN) 2+), and women aged 50–64 years attending routine screening. Sensitivity to CIN2+ was assessed among women with confirmed CIN2+ (colposcopy). Specificity to HPV relative to speculum sampling and overall concordance was assessed among women with negative cytology (routine screening).

**Results:**

The sensitivity of non-speculum sampling for detecting CIN2+ was 83.3% (95% confidence interval [CI] = 60.8 to 94.2) (*n* = 15/18). There was complete concordance among women with positive CIN2+ who had a speculum sample ≤91 days prior to the non-speculum sample (*n* = 12). Among 204 women with negative cytology, the specificity to HPV was 96.4% (95% CI = 92.7 to 98.5), with 96.6% concordant results (κ 72.4%). Seventy-one percent (*n* = 120/170) of women preferred a non-speculum sample for their next screen.

**Conclusion:**

HPV testing on non-speculum clinician-taken samples is a viable approach that warrants further exploration in larger studies. Overall test performance was broadly comparable with that of self-sampling.

## INTRODUCTION

In the UK, around 20% of new cervical cancer diagnoses and 46% of cervical cancer deaths occur in women aged ≥65 years.^1^ Most occur in women who are not screened adequately when aged 50–64 years.^2^ Women aged ≥65 years who are regularly screened have a much lower risk of developing disease compared with those suboptimally screened when aged 50–64 years.^2^ This occurs against a backdrop of falling screening coverage.^3^ The proportion of women screened at least once after reaching 60 years of age declined from 86.4% among women born in 1928– 1931 to 71.3% in women born in 1947–1951 in England.^4^ These data highlight a need to ensure more older women are well screened.

Non-attendance for cervical screening in older women is more likely to be an active decision,^5^ and is associated with perceptions of low risk and low levels of cervical-screening knowledge.^6^ Conventional screening with the speculum can become painful in older women because of musculoskeletal problems and vaginal atrophy.^6^^,^^7^ Women have reported pain during insertion and opening of the speculum, and increased pain during screening after the menopause.^8^

Self-sampling could address these barriers but some women prefer clinician-based screening.^9^ In addition, women worry about taking a self-sample properly and lack confidence in the results.^10^^–^13 Offering a clinician-taken vaginal sample (that is, a vaginal swab taken by a nurse or a doctor) without a speculum (non-speculum clinician sampling) is another possibility; women would have the reassurance of a clinician-taken sample without the discomfort of the speculum. The authors have recently shown that offering both self-sampling and non-speculum clinician sampling substantially increased screening uptake in lapsed attendees aged 50–64 years.^9^

To the authors’ knowledge, test performance of non-speculum clinician sampling has not been reported previously. The study reported here was conducted as a proof-of-concept study to provide early test performance data on this novel approach. The fact that human papillomavirus (HPV) DNA concentration and sensitivity of HPV testing may be lower in older women further underscored the need for non-speculum test performance to be evaluated in older women.^14^^,^^15^ The primary aims of the study were to assess:

- sensitivity of non-speculum samples to high-grade cervical intraepithelial neoplasia (CIN) (that is, ≥grade 2 [CIN2+]);
- relative specificity of non-speculum samples to conventionally taken samples; and
- the concordance of HPV testing on non-speculum samples with matched speculum samples in older women.

**Table:** How this fits in

Speculum use is a significant barrier to cervical screening and can become particularly uncomfortable after the menopause. Self-sampling is an obvious solution but does not appeal to all women. Having a doctor or nurse take a sample without a speculum is another possibility, but test performance has not yet been examined. HPV testing on non-speculum clinician-taken samples was found to have comparable test performance with self-sampling, representing a promising new approach to cervical screening.

The secondary aim was to assess the acceptability of non-speculum sampling in older women.

## METHOD

A prospective, cross-sectional study was conducted in London, UK. Two cohorts of women were recruited:

- a colposcopy population to assess sensitivity to CIN2+; and
- a routine-screening population to assess relative specificity of non-speculum sampling to HPV among women with negative cytology.

Acceptability was assessed in the routine-screening population. During this study, the English cervical screening programme used liquid-based cytology (LBC) with HPV triage (that is, reflex HPV testing if cytology was abnormal).

Ethical approval for the study was gained and all women provided written informed consent prior to participation.

### Study population

For the colposcopy population, two groups of women were recruited from four colposcopy clinics in London between November 2017 and January 2019. The first group comprised women aged ≥50 years, referred with moderate or worse dyskaryosis, or who were HPV positive with a low-grade or borderline cytological abnormality; these were, therefore, either known to be — or likely to be — HPV positive at the time of referral. The second group comprised women aged ≥35 years with CIN2+ previously confirmed on biopsy (histology).

Colposcopy clinic staff identified women and sent study documentation (an invitation letter, patient information leaflet, and HPV information sheet) to them with their appointment letter.

Women in the routine-screening population were recruited from 11 GP practices in East London between August 2017 and March 2018. Those who were eligible were aged 50–64 years and due for routine cervical screening; they were identified through an electronic patient record (EPR) search conducted at each practice and sent an invitation letter, patient information leaflet, and HPV information sheet. Eligible women were also flagged in the EPRs so they could be invited opportunistically (and given study information) if they contacted or consulted the practice.

### Sample collection

All non-speculum samples were collected using a Copan FLOQSwab 552C. In the colposcopy population, these were collected *before* a speculum was inserted and prior to colposcopic examination. In order to provide a speculum HPV result, the next sample collected was either a conventional LBC sample or a cervico-vaginal sample with a speculum using the Copan FLOQSwab 552C. For the latter, sample takers were instructed to ensure they sampled from the cervix and rotated the swab at least four times. In the routine-screening population, all non-speculum samples were collected *before* a speculum was inserted and prior to conventional sample collection.

Written and pictorial instructions on how to collect non-speculum samples (see Supplementary Figure S1) were provided to sample takers. They were not required to touch the cervix with the swab. Conventional samples were collected in line with usual practice (Cervex-Brush^®^ [Rovers^®^ Medical Devices, the Netherlands] in ThinPrep^®^ solution [PreservCyt Solution, Hologic, UK]).

### HPV DNA testing

All study samples were analysed by the Cytology Department at Barts Health NHS Trust, London, UK. HPV testing was performed using Cobas^®^ 4800 HPV Test (Roche Diagnostics GmBH) within 7 days of receipt. Cobas^®^ 4800 is a clinically validated, real-time polymerase chain reaction (PCR) assay that detects 14 high-risk HPV types — types 16, 18, 31, 33, 35, 39, 45, 51, 52, 56, 58, 59, 66, and 68 — in a single analysis. The assay simultaneously tests for human beta-globin as an internal control of sufficient specimen cellularity. Human beta-globin negatives are reported as ‘insufficient’.

All samples were tested for high-risk HPV types. Both non-speculum and speculum flocked swab samples were transported dry at ambient temperature. Swabs were resuspended in 2 mL of PreservCyt solution, vortexed in the original swab tubes for 2 minutes, then processed in the usual manner for HPV testing in accordance with the manufacturer’s protocol. Cervical LBC samples were suspended in ThinPrep solution (PreservCyt Solution, Hologic, UK) in accordance with the manufacturer’s instructions and as per standard practice under the NHS Cervical Screening Programme.

Non-speculum HPV results were not reported to women or their clinicians. Women attending routine screening who tested HPV positive/cytology negative on their conventional sample were referred to colposcopy as a precaution.

### Histopathology reading

Histological reports were obtained from the hospital colposcopy database for cervical biopsies and treatment samples. To ensure a robust classification of true high-grade cervical disease, histology was taken as the lowest grade of pathology if a range was reported (that is, ‘CIN1–2’ was analysed as CIN1), and as the highest grade if multiple grades were reported (that is, ‘CIN2 and CIN3’ was analysed as CIN3).

### Questionnaire

Women attending routine screening completed a short questionnaire^16^ (see Supplementary Box S1) to elicit their views and experiences of non-speculum sampling, along with their future screening preferences. The questionnaire was also used in the publication referenced.

### Statistical analysis

Descriptive analyses were carried out. For analysis of HPV typing, results with positivity for both HPV16 and other high-risk types were categorised as HPV16. Attitudinal items from the questionnaire were dichotomised, and Pearson’s χ^2^ statistic was used to test for differences between non-speculum and conventional sampling.

Cohen’s κ statistic was used to assess concordance between paired samples — that is, a measure of the agreement between two methods in excess of that due to chance. The strength of agreement was judged as poor (<0), slight (0–0.20), fair (0.21– 0.40), moderate (0.41–0.60), substantial (0.61– 0.80), and almost perfect (0.81–1.00).^17^ McNemar’s test was used to assess the relative loss of sensitivity or specificity for non-speculum versus speculum samples. Confidence intervals (CIs) were calculated using exact Clopper–Pearson CIs.^18^

Statistical analyses were conducted using Stata (version 17).

#### Women with CIN2+

The colposcopy population was used to evaluate the sensitivity of non-speculum testing to CIN2+, using a gold standard of histologically confirmed CIN2+. These analyses were restricted to individuals with a non-speculum sample collected before a sample showing CIN2+. The concordance between non-speculum and speculum samples among women with CIN2+ was calculated using the most recent speculum sample collected ≤91 days prior to the non-speculum sample. Sensitivity analyses were conducted for speculum samples collected within 31 days and on the same day. An additional sensitivity analysis excluded women who had had a biopsy or excisional treatment between their speculum and non-speculum samples. If both a conventional and a study speculum (that is, a dry, flocked swab) sample were collected on the same day as the non-speculum sample, the gold-standard conventional sample was used.

#### Women with negative cytology on routine screening

The specificity of non-speculum HPV testing relative to conventional HPV testing among women with negative cytology attending routine screening was evaluated. The main analyses were based on conventional and non-speculum samples collected on the same day. Sensitivity analyses considered tests taken within 14 days; these were conducted for all HPV types combined, and stratified by HPV type (the categories of HPV16 and other high-risk HPV).

#### Women with <CIN2 (colposcopy population)

Concordance between non-speculum and speculum samples among women with <CIN2 in the colposcopy population (including women in whom no biopsy was taken) was assessed. Analysis was restricted to samples that were taken within 91 days of each other, using the speculum sample closest to the non-speculum sample selected.

## RESULTS

**Figure 1.**
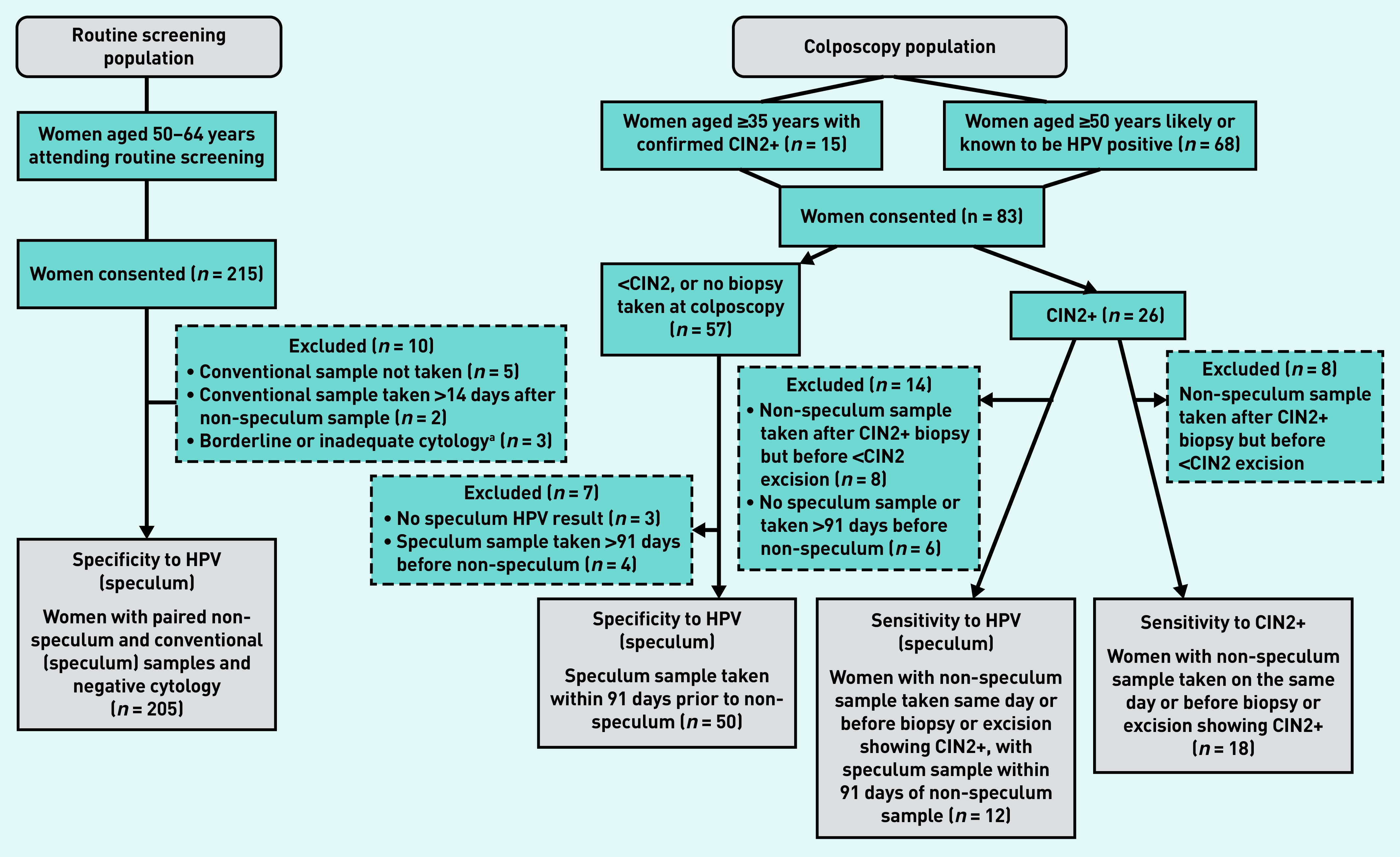
*A flow chart for study participants in the routine screening population and colposcopy population.* *^a^* ***The two women with borderline cytology were HPV negative and are, therefore, not included in the colposcopy population; the third woman had inadequate cytology. CIN = cervical intraepithelial neoplasia. HPV = human papillomavirus.***

Eighty-three women aged 35–70 years were recruited from colposcopy clinics (Figure 1); all but one non-speculum sample was adequate for HPV analysis. Table 1 shows the characteristics of this cohort. Sixty women had a routinely indicated cervical biopsy and/or excision and, of these, 26 had histology showing CIN2+ (Figure 1). Ten women had CIN3 (data not shown).

**Table 1.** Descriptive characteristics of the routine-screening population and colposcopy population participants

**Characteristic**	***n***	**%**
**Routine-screening population**		

**Age, years**		
50–54	55	26.8
55–59	81	39.5
60–64	69	33.7

**Menopausal status^a^**		
Pre-menopausal	5	2.7
Peri-menopausal	9	4.9
Post-menopausal	150	82.4
Not reported/unknown	18	9.9

**Colposcopy population**		

**Age, years**		
35–49	13	15.7
50–54	33	39.8
55–59	22	26.5
60–70	15	18.1

**Reason for referral population study eligibility**		
Confirmed CIN2+ on biopsy	15	18.1
Moderate dyskaryosis on cytology	9	10.8
Severe dyskaryosis on cytology	4	4.8
HPV triage^b^	55	66.3

**Age of women with CIN2+, years**		
35–49	5	27.8
50–54	6	33.3
55–59	3	16.7
60–70	4	22.2

a *Derived from questionnaire data, therefore, the total number of women is 182.*

b *Mild dyskaryosis or borderline changes and HPV positive — 44 women with recent HPV triage, 11 women in follow-up for HPV triage. CIN = cervical intraepithelial neoplasia. HPV = human papillomavirus.*

For the routine-screening study, invitation letters were sent to 1437 women identified through the EPR search. In total, 215 women were recruited and provided a non-speculum sample (Figure 1). Pain on insertion of the speculum led to the exclusion of seven women: two had conventional samples collected >14 days after the non-speculum sample and five had no conventional sample taken. In addition, three women with non-negative cytology results were excluded (inadequate: *n* = 1, borderline: *n* = 2), resulting in 205 women with paired samples taken within 14 days that were available for analysis (Figure 1); of these, 204 women had their speculum and non-speculum samples taken on the same day.

### Women with CIN2+

Twenty-six women had CIN2+ on histology; of these, 18 had a non-speculum sample collected on the same day or before a biopsy or excision showing CIN2+ (Figure 1). Fifteen women tested HPV positive on non-speculum testing (sensitivity = 83.3%, 95% CI = 60.8 to 94.2). Twelve women had a speculum sample collected within 91 days prior to the non-speculum sample; there was complete concordance in their results, with 11 women positive on both tests, and one woman negative on both tests (Table 2). One woman who was positive on both tests had a biopsy or excisional treatment between the speculum and non-speculum tests. Detailed sensitivity analyses are shown in Supplementary Table S1.

**Table 2.** Paired speculum and non-speculum sample results among women with CIN2+ on biopsy or excision

**Speculum sample**	**Non-speculum sample**		
	**HPV positive, *n* (%)**	**HPV negative, *n* (%)**	**Total, *n* (%)**
HPV positive	11 (91.7)	0 (0.0)	11 (91.7)
HPV negative	0 (0.0)	1 (8.3)	1 (8.3)
Total	11 (91.7)	1 (8.3)	12 (100.0)

*CIN = cervical intraepithelial neoplasia. HPV = human papillomavirus.*

### Women with negative cytology at routine screening

Of the 204 women whose conventional and non-speculum samples were taken on the same day, 197 had concordant results (96.6%); 10 women were positive on both tests (Table 3). The specificity was 96.4% (95% CI = 92.7 to 98.5%) for non-speculum sampling compared with conventional sampling (κ 72.4%) (data not shown). The McNemar’s test χ^2^ statistic was 7.00 (*P* = 0.02), showing a statistically significant difference in the agreement between conventional and non-speculum tests — all seven discordant results (3.4%) were positive on non-speculum samples and negative on conventional samples (Table 3), corresponding to a relative excess in HPV positivity of 70.0%.

**Table 3.** Paired speculum and non-speculum sample results among women with negative cytology, who had speculum and non-speculum samples collected on the same day

**Speculum sample**	**Non-speculum sample**		
	**HPV positive, *n* (%)**	**HPV negative, *n* (%)**	**Total, *n* (%)**
HPV positive	10 (4.9)	0 (0.0)	10 (4.9)
HPV negative	7 (3.4)	187 (91.7)	194 (95.1)
Total	17 (8.3)	187 (91.7)	204 (100.0)

*HPV = human papillomavirus.*

One additional woman who had the non-speculum and conventional samples taken on different days, but within 14 days, was HPV negative on both tests (data not shown). Two women were protocol violations and collected the non-speculum samples themselves; both were HPV negative on both samples (data not shown).

Although numbers were small, all three women who had HPV16 detected on their speculum sample also had HPV16 detected on their non-speculum sample (see Supplementary Table S2).

### Women with <CIN2

In the colposcopy population, 34 women had a biopsy or excision with <CIN2 and 23 had no biopsy. Of these 57 women, three had no speculum HPV result and four had a speculum sample collected >91 days prior to their non-speculum sample; this resulted in 50 women whose results could be included in the analysis (Figure 1). Table 4 shows matched speculum and non-speculum results. Results were concordant for 46 out of 50 (92.0%) women, with 41 (82.0%) women testing positive on both samples. Of the four with discordant results, all tested positive on speculum samples but negative on non-speculum samples (κ 0.67; *P*<0.001; McNemar’s test χ^2^ 3.0; *P* = 0.046). Sensitivity analyses are shown in Supplementary Table S3.

**Table 4.** Paired speculum and non-speculum sample results among women with <CIN2 on biopsy or excision, or who attended colposcopy and no biopsy was taken

**Speculum sample**	**Non-speculum sample**		
	**HPV positive, *n* (%)**	**HPV negative, *n* (%)**	**Total, *n* (%)**
HPV positive	41 (82.0)	4 (8.0)	45 (90.0)
HPV negative	0 (0.0)	5 (10.0)	5 (10.0)
Total	41 (82.0)	9 (18.0)	50 (100)

*CIN = cervical intraepithelial neoplasia. HPV = human papillomavirus.*

### Acceptability of non-speculum clinician-taken sampling

Table 5 details the results from the women in the routine-screening population who returned a questionnaire (*n* = 182/215; 84.7%); item non-response was low (<7.0%). A majority of women (*n* = 150/164; 91.5%) reported being post-menopausal (Table 1); not all women responded to all questions. Most women found both the taking of non-speculum and conventional samples to be an ‘excellent/good’ overall experience (90.1% and 73.3% respectively; *P*<0.001), but discomfort was higher for conventional samples (76.9% versus 36.5% [mild/quite a lot/severe discomfort]; *P*<0.001) (Table 5). Most women (*n* = 133/171; 77.8%) preferred the non-speculum sample over the conventional sample, and two-thirds (*n* = 120/170; 70.6%) reported that they would prefer a non-speculum sample for their next screen.

**Table 5.** Questionnaire responses from routine-screening participants (*n* = 182) who had both speculum and non-speculum samples collected

**Experience**	**Non-speculum sample, *n* (%)^a^**	**Conventional sample, *n* (%)^a^**	**Pearson’s** χ**^2^ test, *P*-value**
**Overall experience of test**			
Excellent/good	164 (90.1)	132 (73.3)	<0.001
Fair/poor	18 (9.9)	48 (26.7)	
Missing	0	2	
**Discomfort**			
None	115 (63.5)	42 (23.1)	<0.001
Mild/quite a lot/severe	66 (36.5)	140 (76.9)	
Missing	1	0	
**Embarrassment**			
Not at all	128 (70.7)	121 (66.5)	0.45
Mildly/fairly/very	53 (29.3)	61 (33.5)	
Missing	1	0	

**Preferences**			
**Test preference**			
Non-speculum	133 (77.8)		
Conventional speculum	14 (8.2)		
No preference	24 (14.0)		
Missing	11		
**Future preference**			
Non-speculum	120 (70.6)		
Conventional speculum	17 (10.0)		
No preference	33 (19.4)		
Missing	12		

a *Reported as the percentage of responders to that question.*

## DISCUSSION

### Summary

The sensitivity of non-speculum samples for detecting CIN2+ was good (83.3%). Non-speculum samples showed high concordance with conventional samples for detecting HPV in routine-screening and colposcopy populations. In routine screening, HPV detection was 3.4% higher in non-speculum samples compared with conventional samples, presumably due to the detection of vaginal HPV infections. Non-speculum sampling had high acceptability, with >90% of women reporting it to be an excellent or good experience.

### Strengths and limitations

The main strength of this study is that, to the authors’ knowledge, it is the first to examine test performance of non-speculum clinician sampling for HPV testing. In addition, both routine-screening and colposcopy populations were included, which enabled test performance to be assessed in women with different underlying prevalence of disease. Non-speculum samples were collected and processed in a setting that is analogous to the setting that would be used if non-speculum testing was offered as part of the national screening programme. Only one of the 298 non-speculum samples was inadequate for analysis.

Important limitations include the relatively small number of CIN2+ cases (*n* = 18) and the fact that non-speculum and speculum samples were not always collected on the same day, with some collected after biopsy. HPV clearance in women aged 60–89 years has been reported to be as high as 37% over an average of 3.5 months;^19^ similarly, punch biopsy can shorten the time to clearance.^20^ These factors may have resulted in underestimates of non-speculum sample sensitivity to CIN2+. Conversely, the use of a referral population comprising women with cytological abnormalities may have led to overinflated estimates of sensitivity to CIN2+, given that their results are more likely to be HPV positive than those of women undergoing routine screening.

It is worth highlighting that clinical test performance is known to vary with the type of HPV assay used.^21^ As such, the generalisability of the results may be limited as they were based on a single PCR-based HPV assay, which is known to have greater sensitivity than non-PCR-based assays. However, this study was not designed to be definitive but, rather, to provide confidence that test performance was not unexpectedly low before offering it in a randomised controlled trial.^9^

### Comparison with existing literature

As non-speculum clinician sampling has not previously been evaluated to the authors’ knowledge, the findings reported here can only be compared with that of self-sampling. The sensitivity to CIN2+ for non-speculum samples (83.3%; 95% CI = 60.8 to 94.2) was lower than the reported sensitivity of vaginal self-samples from 130 women with CIN2+ in Scotland (94.6%; 95% CI = 90.7 to 98.5), but the 95% CIs overlap considerably.^22^

The study presented here found almost identical concordance to that of a Dutch study^23^ of self-samples and cervical (speculum) samples from 2049 women attending routine screening (96.6% versus 96.8%, respectively). The excess HPV positivity rate (relative excess: 70%, absolute excess: 3.4%) associated with non-speculum sampling versus conventional sampling was between what has been reported in two self-sampling studies conducted in routine-screening populations in the Netherlands (relative excess: 30%, absolute excess: 1.6% among those aged 49–63 years)^23^ and Scotland (relative excess: 138%, absolute excess: 3.9% in those aged 50–59 years).^22^ Notably, the routine-screening population used in the study presented here was restricted to women with negative cytology.

Participants’ reports of positive experiences with non-speculum sampling chimes with the authors’ qualitative acceptability work,^7^ which found that older women would welcome the opportunity to be screened using the non-speculum clinician-sampling approach.

### Implications for research and practice

Screening non-attendance remains an important risk factor for cervical cancer. Although this study focused on older women, non-speculum clinician sampling could appeal to women of any age who dislike the speculum but prefer a clinician to take their sample. Self-sampling does not appeal to all women who dislike speculum-based screening,^9^ which highlights a need for alternative speculum-free screening approaches.

In a related study,^9^ the authors showed that offering non-speculum and self-sampling to older women whose screening attendance had lapsed substantially increased uptake by 17% (95% CI = 11.3 to 22.7; *P*<0.001) — from 14% to 31% — with 23% of screened women opting for non-speculum sampling. The study also found that having a choice of screening tests was important to women. The fact that conventional samples could not be collected from 3% of women attending routine screening (all of whom provided an adequate non-speculum sample) reinforces the clinical need for this approach. It also provides an example of the clinical context in which non-speculum sampling could be used. Such women are highly motivated to be screened, but are likely to be underscreened unless speculum-free screening options are provided.

A high proportion of women attending routine screening indicated that they preferred non-speculum clinician sampling over conventional sampling, and would prefer it for future screening tests; this suggests that many regular attendees could switch to non-speculum sampling if it is introduced. This has important workload and cost implications: swab samples require manual laboratory processing and women who test positive for HPV would require a follow-up appointment. In addition, the HPV positivity rate was 3.4% higher in non-speculum samples. This has the same clinical implications as self-sampling: a follow-up cervical screen to identify which women require colposcopy, with the knowledge that some of these women would be HPV negative on a speculum sample.

Self-sampling is now widely accepted as being clinically non-inferior to conventional clinician-taken samples.^21^^,^^24^^,^^25^ Although the authors of the study presented here found the sensitivity of non-speculum sampling to CIN2+ to be slightly lower than for self-sampling, the CIs overlapped, and no women in the routine-screening population were speculum positive/non-speculum negative. Given that the two collection methods are technically the same, it would be surprising if clinical performance was inferior to self-sampling. The non-speculum approach could easily be implemented into current clinical practice, potentially dovetailing with self-sampling.

Further potential advantages of non-speculum sampling over self-sampling include that the relationship between the sample taker and the woman is preserved, providing an opportunity to discuss cervical screening and general gynaecological health issues, as well as emphasising the importance of follow-up. Non-speculum clinician sampling removes the risk of false negatives associated with women self-sampling from the incorrect area (for example, superficial genitalia). Finally, it provides a speculum-free option for women who may find it difficult to collect a self-sample because of learning difficulties, physical disabilities, or mobility issues. The approach could be a promising addition to cervical screening and warrants further exploration in larger studies. In particular, larger paired sample studies with clinical outcomes across all screening age groups are needed to better define test performance.

## Data

Anonymous datasets can be made available upon request.
